# Tolerance and Safety of an Anti-Regurgitation Formula Containing Locust Bean Gum, Pre-, and Postbiotics: A Multi-Country Multi-Center Prospective Randomized Controlled Study in Infants with Regurgitation

**DOI:** 10.3390/nu16060899

**Published:** 2024-03-20

**Authors:** Silvia Salvatore, Viktoriia Klymenko, Yuliia Karpushenko, Maria Durczak-Hilleman, Andrii Loboda, Viktoriia Petrashenko, Wiesław Olechowski, Gianluca Lista, Fabio Meneghin, Sonia Amodio, Anke Bongers, Thomas Ludwig, Yvan Vandenplas

**Affiliations:** 1 Pediatric Department, Hospital “F. Del Ponte”, University of Insubria, 21100 Varese, Italy; 2 Propaedeutics of Paediatrics No. 2, Communal Nonprofit Enterprise “City Children’s Clinical Hospital # 19” of Kharkiv City Council, 61051 Kharkiv, Ukraine; klymenkoviktoriia@gmail.com (V.K.); juli588k@gmail.com (Y.K.); 3 EPOKA Niepubliczny Zaklad Opieki Zdrowotnej Piotr Chodkiewicz Sp. z o.o., 88-400 Znin, Poland; mariahillemann@o2.pl; 4 Department of Pediatrics, Sumy State University, 40000 Sumy, Ukraine; fafik1313@ukr.net (A.L.); v.petrashenko@med.sumdu.edu.ua (V.P.); 5 Alergo-Med Specjalistyczna Przychodnia Lekarska Sp. z o.o., 33-100 Tarnow, Poland; w.olechowski@gmail.com; 6 SC Neonatologia e Terapia Intensiva Neonatale, Ospedale dei Bambini “V. Buzzi”, 20154 Milano, Italy; gianluca.lista@asst-fbf-sacco.it (G.L.); fabio.meneghin@asst-fbf-sacco.it (F.M.); 7 Specialised Nutrition, Danone Nutricia Research, 3584 CT Utrecht, The Netherlands; sonia.amodio@danone.com (S.A.); anke.bongers@danone.com (A.B.); thomas.ludwig@danone.com (T.L.); 8 KidZ Health Castle, UZ Brussel, Vrije Universiteit Brussel (VUB), 1090 Brussels, Belgium; yvan.vandenplas@uzbrussel.be

**Keywords:** regurgitation, gastroesophageal reflux, thickened formula, functional gastrointestinal disorder, locust bean gum

## Abstract

This multi-center prospective randomized controlled trial was a tolerance and safety study investigating the thickener locust bean gum (LBG) in infants with regurgitation, to support the re-evaluation of the safety of LBG in infant formula. The primary objective was to demonstrate that after an 8-week intervention, stool consistency was not inferior (i.e., was not looser or more watery) in infants fed an anti-regurgitation (AR) formula containing LBG vs. the stool consistency of infants fed with an unthickened control formula. A total of 103 full-term infants with regurgitation were randomized to the test or control formula. The test formula contained LBG (0.4 g/100 mL), short-chain galacto-oligosaccharides, and long-chain fructo-oligosaccharides (scGOS/lcFOS; 9:1; 0.4 g/100 mL) and postbiotics and the control formula contained scGOS/lcFOS (0.8 g/100 mL), the same amount of postbiotics, and did not contain LBG. The average stool consistency score at the 8th intervention week was the primary outcome parameter. Secondary outcome parameters were stool consistency at other timepoints, stool frequency, Infant Gastrointestinal Symptom Questionnaire (IGSQ) score, growth, (serious) adverse events ([S]AEs), regurgitation severity, and infant well-being. Overall, the infants were 36.9 ± 12.9 [mean ± SD] days old, 62.7% girls in the test, and 50.0% girls in the control group. The primary analysis showed that the test group did not have looser or more watery stools than the control group. IGSQ sum scores decreased comparably in both groups. The frequency of regurgitation was significantly lower in the test group compared to the control group (mixed model repeated measurement, *p* ≤ 0.028) and parent-reported well-being scores were favorable. Adequate growth was observed in both groups. Both products were well-tolerated and safe and the AR formula with LBG was efficacious in reducing regurgitation frequency. This study provides further evidence for the dietary management of regurgitation by LBG-containing formulae in infants who are not exclusively breastfed, and the reassurance it can bring to parents.

## 1. Introduction

Regurgitation is a common functional gastrointestinal (GI) disorder in infants which frequently causes parental concern and unnecessary use of medication [1,2,3]. Regurgitation has been reported as also being associated with symptoms of infant colic and functional constipation [4,5,6]. In general, children with functional GI disorders have been reported to have more frequently functional GI symptoms and pain disorders later in life [7,8].

The management recommendations of regurgitation for all infants emphasize parental reassurance, education, and nutritional advice (i.e., feeding volumes and intervals). For infants that are not exclusively breastfed, even if suspected of gastro-esophageal reflux [9], this may also include the use of thickened infant formula [9,10,11]. The use of medication, such as proton pump inhibitors, is generally not recommended in infants with regurgitation [10,12].

Locust bean gum (LBG, otherwise known as carob bean gum) is a fiber that is widely used as a natural thickening agent in anti-regurgitation (AR) infant formula. In vitro and in vivo data have shown that LBG remains intact in the stomach, resists digestion, and is not absorbed in the human gut. In addition, LBG has a lower impact on the glucose response and has a much higher viscosity than thickeners based on starch compounds, allowing a smaller quantity of thickener to provide the same result [2]. AR formulae containing LBG have been demonstrated to be safe and effective in reducing the frequency and volume of regurgitation in numerous clinical studies of infants with regurgitation [13,14,15,16,17,18]. In addition, infant formulae containing locust bean gum have been used worldwide without reported safety concerns [19].

Recently, a large (*n* = 2604 infants) open, one-month, prospective, observational study of an LBG-thickened (0.4 g/100 mL) formula showed good tolerance and multiple beneficial clinical effects in infants with regurgitation [14]. Regurgitation frequency and volume decreased significantly, with total resolution of regurgitation episodes in half (48%) of the infants within one month. A significant decrease in the duration of crying and episodes of gas as well as an improvement in quality of life were also observed. Stool frequency increased and stool consistency softened but remained within the physiologic range [14].

In a 4-week double-blind, randomized, controlled trial (DBRCT) an AR (Test) formula containing LBG (0.4 g/100 mL), postbiotics, and short-chain galacto-oligosaccharides (scGOSs) and long-chain fructo-oligosaccharides (lcFOSs) (0.4 g/100 mL, ratio 9:1) was compared to an AR formula with LBG (0.4 g/100 mL) and postbiotics, but without prebiotics, in 182 infants [17]. The primary outcome was based on the Infant Gastrointestinal Symptom Questionnaire (IGSQ) sum score, that considers stool frequency and consistency, regurgitation/vomiting, crying, fussiness, and flatulence. IGSQ scores improved significantly within 1 week, particularly in infants with more severe GI symptoms (baseline IGSQ score ≥ 35), when receiving the test formula vs. the control formula. Stool characteristics were comparable between groups [17].

In another multi-center observational prospective trial which enrolled 190 infants, the same test formula containing LBG, prebiotics, and postbiotics was well tolerated and, at three months of intervention, there was no significant increase in the number of infants with diarrhea. Moreover, the test formula significantly decreased regurgitation and crying severity and the number of infants with constipation. In addition, infant well-being and sleep quality improved, as reported by the parents [18].

The current randomized controlled study has been designed to address the European Food Safety Authority (EFSA) call for data to support the re-evaluation of the safety of LBG in infant formulae [20]. The primary study objective was to demonstrate that in infants with regurgitation, the stool consistency was not inferior (i.e., not looser or more watery) in those consuming an infant formula containing LBG as compared to the stool pattern of infants fed the control formula without LBG. The study confirmed the primary objective and showed that both study formulae were well-tolerated and safe. Infants consuming the AR formula with LBG did not have looser or more watery stools compared to those who consumed the formula without LBG.

## 2. Materials and Methods

### 2.1. Design

This was a multi-country multi-center prospective randomized controlled tolerance and safety study (ClinicalTrials.gov identifier: NCT04042454). The study was performed in compliance with the statement of the Declaration of Helsinki and approved by the Ethics Committees (protocol number EBB18TA19425; first approval 29 January 2019).

### 2.2. Subjects

The recruitment of subjects took place in 13 pediatric centers in Poland, Ukraine, and Italy from December 2019 to November 2021. Only those parents who autonomously decided to fully formula-feed their infant were contacted and informed about the study. Written informed consent was obtained from parents before the screening procedures. Infants were eligible when they were singleton, term-born (≥37 and ≤42 weeks gestational age), aged ≥ 21 days and ≤63 days, and fully formula-fed for at least one week prior to study participation. The infants’ diagnosis of regurgitation was based on adapted Rome IV criteria [21,22], including the presence of ≥2 regurgitation episodes per day for ≥7 days prior to screening and the absence of retching, hematemesis, aspiration, apnea, failure to thrive, feeding or swallowing difficulties, or abnormal posturing. Exclusion criteria were low birth weight for gestational age and sex (<10th percentile compared to the intergrowth standards [23]), suspicion or diagnosis of gastroesophageal reflux disease and/or cow’s milk protein allergy, gastrointestinal infection, congenital conditions and/or GI malformation, presence of any other GI symptoms and a known allergy to the formula’s ingredients. Infants were also excluded if they used systemic antibiotics, prokinetics, proton pump inhibitors, or other anti-reflux medication, a thickened formula, thickening supplements and/or complementary feeding, and if they participated in another intervention study.

### 2.3. Intervention

Participants were randomized (1:1 ratio) with a computer randomization system to the test AR formula group or to an unthickened control formula group. Randomization was performed with permuted block randomization stratified per center. A study-independent statistician generated the allocation sequence. Intervention took place for at least 8 weeks, with a maximum cut-off when the infant reached 17 weeks of age. Both infant formulae were commercially available, cow milk-based, and nutritionally complete powders manufactured by Danone Nutricia. The test product contained LBG (0.4 g/100 mL), short-chain galacto-oligosaccharides and long-chain fructo-oligosaccharides (scGOS/lcFOS; 9:1; 0.4 g/100 mL), and 26% fermented formula with postbiotics derived from the Lactofidus^TM^ fermentation process, including 3′-Galactosyllactose. The unthickened control product contained the same amount of non-digestible carbohydrates as the test AR product but was based on scGOS/lcFOS (0.8 g/100 mL) and did not contain LBG. The levels of the other components, including the postbiotics, were similar in the test and control formula (Table S1). As there was a small chance that the nature of the study product (thickened or unthickened formula), would be noticed by the parents, the study was not labelled as a double-blind study. However, all measures and procedures were followed as they would be for a double-blind study.

### 2.4. Outcome Measures

The Brussels Infant and Toddler Stool Scale (BITSS) [24,25] was used to assess stool consistency, defining stool consistency in 4 different categories: watery (score 1), loose (score 2), formed (score 3), or hard (score 4). Parents were instructed to score stool consistency daily in a paper diary, throughout the first 8 weeks of intervention and in the week prior to the last study visit when the infant was 17 weeks old. Daily scores from the same subject were averaged for each week when at least 3 days per diary week were completed. At baseline, stool consistency scores were registered retrospectively (parent’s recall based on 24 h prior to Baseline (Visit 1)) and prospectively in a diary for 24 h after Visit 1, prior to starting to feed with the study product. The average of the data collected during the 24 h before and after Visit 1 was used as baseline score. The average stool consistency score at the 8th intervention week was the primary outcome parameter to investigate non-inferiority. In addition, the percentage of each stool consistency category, the occurrence of ≥3 watery stools per day and the occurrence of hard stools (≤2 defecations per week) were analyzed as secondary outcomes.

Other secondary outcomes were infant growth, diarrhea incidence (reported as adverse events [AEs]), the 13-item Infant Gastrointestinal Symptom Questionnaire (IGSQ) [26] (as total and individual item scores), and regurgitation frequency and volume (based on parent-reported diaries). The IGSQ was administered by a trained blinded assessor at baseline (Visit 1), week 2 (Visit 2), week 4 (Visit 3), week 8 (Visit 4) and during the last visit when the infant was 17 weeks old (Visit 5). The IGSQ consists of 13 items, each scored on a 1–5 point scale, with higher scores pointing to a higher GI symptom burden.

The IGSQ sum score varied from 13 to 65 [26]. At the final study visit, parents completed an evaluation form including questions on the study product and on the general well-being of their infant (questions scored on a 10-point scale grading from 1 = not at all satisfied to 10 = very satisfied). Exploratory outcomes included parent-reported recording of infant crying and blood mineral status (optional assessment, only in the event that parents gave separate consent). Samples of infant’s stool were also collected, at baseline and at the age of 17 weeks, to assess gut microbiota composition and function. The stool samples have yet to be fully analyzed and are beyond the scope of the present manuscript. During each study visit, investigators measured the subject’s anthropometrics and registered AEs and the use of concomitant medication.

### 2.5. Statistical Analyses

Assuming no difference between the two study groups, 35 evaluable infants per group were deemed required to achieve 90% power to demonstrate non-inferiority in stool consistency in the test vs. the control group (α = 0.05; non-inferiority margin of −0.5). Anticipating a maximum non-evaluability rate of ~30% meant that 100 infants needed to be randomized. A predefined interim analysis of GI tolerance and safety data was performed and the results were evaluated by an independent data monitoring committee. The committee did not identify safety concerns and recommended study continuation without modification.

The per-protocol (PP) population was the main population for the primary analysis and was defined prior to database lock and unblinding. The analyses were repeated on the all-subjects-randomized (ASR) population as supportive analyses. The non-inferiority of the test vs. the control group was examined by analyzing whether the lower bound of the 2-sided 90% confidence interval (CI) of the difference in the average stool consistency score at week 8 lay entirely above the predefined non-inferiority margin of −0.5 at 8 weeks of intervention. A linear mixed-model repeated measurement (MMRM) was used to analyze the data. The MMRM considered study group, categorical time, interaction between study group and time, baseline stool consistency score, and interaction between baseline stool consistency score and time as fixed effects and site as a random effect, fitting separate unstructured covariance matrices for the study group to address heteroscedasticity [27]. For secondary outcome parameters, i.e., the percentage of individual stool consistency categories, stool frequency, and IGSQ item scores, differences between groups were analyzed using a Mann–Whitney test. A Miettinen and Nurminen score test was used to compare groups for the risk difference of having ≥1 day per week with ≥4 regurgitations. The IGSQ sum score and regurgitation frequency were compared between groups using linear MMRMs. For the last outcome parameters, the MMRM considered study group, categorical time, interaction between study group and time, and baseline values of the specific parameter as fixed effects and site as random effect. SAS Life Science Analytics Framework version 5.3 (SAS Institute Inc., Cary, NC, USA) was used for all statistical analyses.

## 3. Results

### 3.1. Subjects

**Figure 1:**
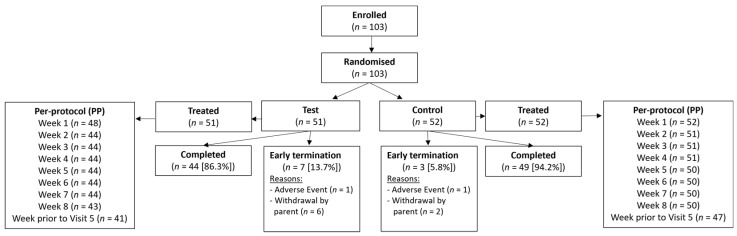
Subject flow.

Between December 2019 and November 2021, 103 infants were randomized to the test group or the control group (Figure 1). Except for a higher percentage of girls in the test group compared to the control group (62.7% vs. 50.0%), demographics were comparable between the study groups. In the overall study population, subjects had an average age of 36.9 ± 12.9 days (mean ± SD) at baseline and a duration of regurgitation prior to inclusion that was 25.6 ± 11.4 days (Table S2). None of the participants received any medication to treat regurgitation. There were seven early terminations (13.7%) in the test group, and three (5.8%) in the control group (no significant difference). The reasons for early termination were an AE (cow’s milk allergy) for 1 subject in each study group and withdrawal by parent (*n* = 6 in test, *n* = 2 in control group).

The per-protocol (PP) population was defined on a weekly level based on blinded data during a data review meeting that took place before database lock and unblinding. The PP population consisted of infants without major violations of the eligibility criteria who were only fed with the study product during the intervention (i.e., no commercial infant formula and no complementary feeding), who did not use systemic antibiotics and for whom at least one post-baseline stool consistency score was available.

### 3.2. Primary Outcome Results

**Figure 2:**
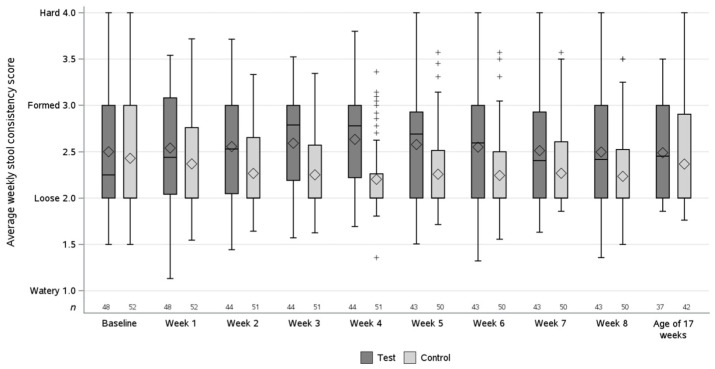
Average stool consistency scores per intervention week and at the age of 17 weeks (visit 5) in the per-protocol population. The box is for the first to the third quartile, with the line inside the box for the median. The diamond refers to the mean. The whiskers extend to the most extreme value within 1½ times the box height around the box. More extreme values are marked separately with the plus sign (+). *n* = no. of subjects in test (dark grey) and control (light grey) group per intervention week.

The average stool consistency scores per intervention week are shown in Figure 2. The non-inferiority of the average stool consistency score at 8 weeks of intervention was shown for the test vs. the control group in both the PP population (difference in estimated means of 0.17, 90% CI [0.07; 0.28]) and the ASR population (difference in estimated means of 0.17, 90% CI [0.07; 0.28]). For both study populations, the lower bound of 90% CI of the difference in estimated means was greater than the prespecified non-inferiority margin of −0.5.

### 3.3. Secondary Outcome Results

As the PP population was the predefined population of interest, the results are presented for this study population. Nonetheless, the results were confirmed for the ASR population.

**Figure 3:**
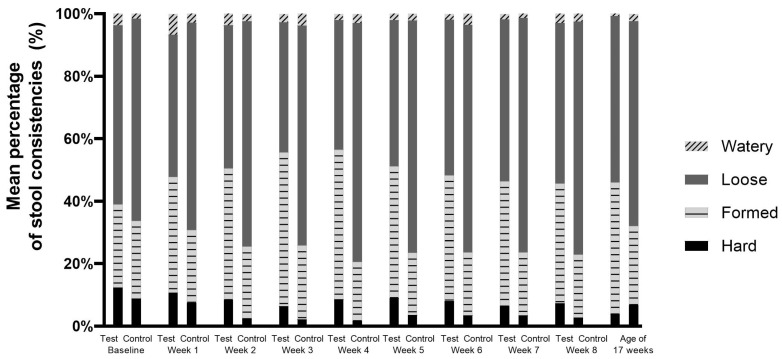
Mean percentage of stool consistencies per intervention week and at the age of 17 weeks (Visit 5) in the per-protocol population.

In both study groups, low percentages of stools were reported to have a watery or hard consistency (Figure 3). In the control group, significantly looser and fewer formed stools were reported compared to the test group (Mann–Whitney U test, *p* ≤ 0.009 for formed stools and *p* ≤ 0.007 for loose stools, up to week 8). There were no statistically significant differences in the percentages of watery stools between the two groups. Statistically significantly more hard stools were observed in the test group vs. the control group at week 2 (8.4 vs. 2.5%; *p* = 0.003) and week 4 (8.7 vs. 1.8%; *p* = 0.002). No statistically significant differences were observed at the other intervention weeks. In both study groups, the percentage of hard stools was not higher at any timepoint compared to the baseline values (Figure 3).

The occurrence of ≥three watery stools per day was low (maximum per intervention week of *n* = three subjects in the test group and *n* = two subjects in the control group; PP population), without statistically significant differences between groups at any intervention week. Hard stools in ≤two defecations per week were not reported. Stool frequency was comparable between study groups, except for small but statistically significant differences in daily stool frequency in the test group vs. the control group at intervention week 2 (median; [Q1; Q3]; test: 2.9 [1.9; 3.3]; control 2.0 [1.3; 3.0]), week 3 (test: 2.6 [1.9; 3.3]; control 2.0 [1.4; 2.7]) and week 4 (test: 2.6 [1.9; 3.1]; control 2.0 [1.1; 2.5]).

The IGSQ sum score decreased from 31.8 ± 8.7 (mean ± SD) at baseline to 25.5 ± 6.5 after 2 weeks of intervention in the overall study population. After 4 weeks of intervention, the average score was 23.5 ± 6.3, followed by a further decrease to 19.2 ± 4.4 at the infant’s age of 17 weeks. There was no significant difference between groups at any timepoint. Similarly, most IGSQ item scores (Table S3) were not significantly different between study groups, except for differences of IGSQ stooling, item 1, and spitting up/vomiting, item 3. The IGSQ item 1 score, regarding the frequency of passing a hard stool, was statistically significantly higher in the test group vs. the control group at Visit 2 (median [Q1; Q3], test: 1 [1; 3]; control: 1 [1; 1]; Mann–Whitney U test, *p* = 0.008). No statistically significant differences were observed during the other visits. The IGSQ item 3 score, regarding the frequency of spitting up/vomiting, was statistically significantly lower in the test group vs. the control group at most study visits (median [Q1; Q3], Visit 3 test: 2 [1; 3]; control: 3 [2; 3]; *p* = 0.001; Visit 4 test: 1 [1; 2]; control: 2 [1; 3]; *p* < 0.001; and Visit 5 test: 1 [1; 2]; control: 2 [2; 3]; *p* = 0.004) and almost reached statistical significance at Visit 2 (test: 2 [2; 3]; control: 3 [2; 3], *p* = 0.055).

**Figure 4:**
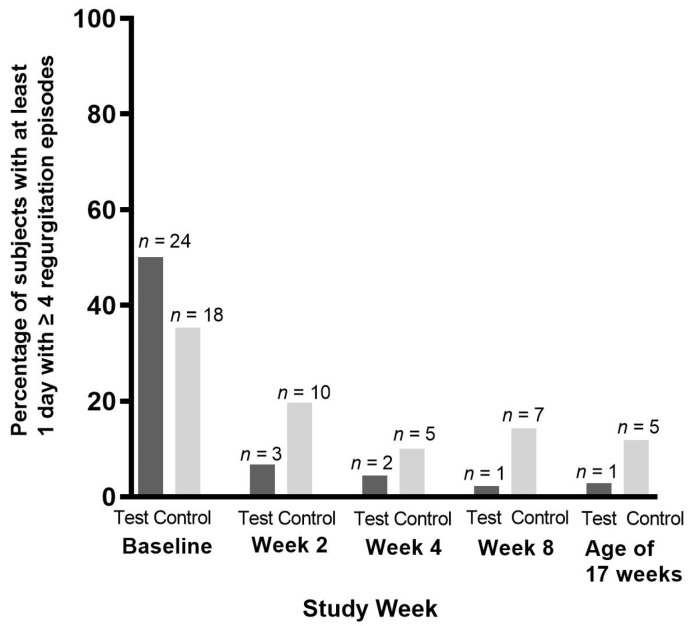
Occurrence of ≥four episodes of regurgitation per day, per intervention week and at the age of 17 weeks (Visit 5) in the per-protocol population.

At baseline, the frequency of regurgitation was higher in the test group compared to the control group (median [Q1; Q3], test: 3.5 [2.0; 5.0]; control: 3.0 [2.0; 4.0] episodes per day; Mann–Whitney U test, *p* = 0.046). Statistical modeling by means of MMRM accounting for this baseline difference showed a statistically significantly lower regurgitation frequency in the test group vs. the control group at every post-baseline timepoint (estimated marginal means [standard error (SE)]: Visit 2: −0.55 [0.25], *p* = 0.028; Visit 3: −0.67 [0.24], *p* = 0.007; Visit 4: −0.78 [0.22], *p* < 0.001; Visit 5: −0.80 [0.28], *p* = 0.006). Similarly, the proportion of subjects with at least 1 day with ≥four regurgitations was lower in the test group at each post-baseline timepoint (Figure 4). Within 2 weeks, the number of subjects with ≥four regurgitation episodes per day decreased by 86.4% in the test vs. 44.5% in the control group (week 2, test group: *n* = 3 [6.8%] vs. control group: *n* = 10 [19.6%]). A statistically significant difference between groups was observed at week 8 (test: *n* = one [2.3%]; control: *n* = seven [14.3%], Miettinen and Nurminen score test, *p* = 0.043), but not at the other timepoints. Regurgitation volume was not statistically significantly different between study groups.

The parental satisfaction with both study products was high, i.e., scoring higher than 7.9. In response to the question whether the parent thought that the infant formula improved the overall well-being of their baby, parents in the test group scored (mean ± SD) 8.3 ± 2.0, compared to 8.1 ± 1.9 in the control group. On the question whether the parent thought the formula helped to reduce the regurgitation of their baby, scores were 8.9 ± 1.8 for the test and 7.5 ± 2.8 for the control group. In addition, both crying frequency and duration were low in both study groups. The parents of four subjects gave consent to have a blood sample collected from their infant. Given this very small sample size, no statistical analyses on mineral status were performed.

There were no significant differences between study groups for mean weight-for-age, length-for-age, weight-for-length, and head circumference-for-age WHO z-scores. Values were in line with the normal ranges referred to by the international WHO growth standards [28].

### 3.4. Adverse Events and Concomitant Medication

The total incidence of (S)AEs was comparable in both study groups (*n* = 12 [23.5%] in the test vs. *n* = 11 [21.2%] in the control group). Most AEs were reported within the system organ class (SOC) infections and infestations and SOC gastrointestinal disorders, with similar type and intensity of the reported events in both groups. In each group, there were two reports of mild diarrhea (3.9% in the test group vs. 3.8% in the control group) and one report of mild flatulence (2.0% in the test group vs. 1.9% in the control group). Abdominal pain (moderate in severity) was reported in two subjects (3.8%) in the control group and was not reported in the test group. No AE constipation was reported. There were two serious AEs (SAEs) in two subjects in each study group. All SAEs were in the SOC infections and infestations and were assessed as not related to the study product by the investigators. No clinically relevant differences between the study groups were observed in the use of concomitant medications and nutritional supplements.

## 4. Discussion

This randomized controlled study demonstrated that the studied AR formula containing LBG combined with pre- and postbiotics did not lead to more loose or watery stools as compared to the control formula containing pre- and postbiotics, but without the LBG thickener. Both formulae were well-tolerated and safe and supported adequate growth in infants with regurgitation. In addition, the AR formula was efficacious in reducing regurgitation frequency.

In each study group, stool consistency was within the physiological range and the occurrence of watery or hard stools was relatively low. At weeks 2 and 4, there were significantly more hard stools in the test group compared to the control group, but this difference was not observed at the other study weeks and none of the infants with hard stools had a defecation frequency ≤ two per week. In addition, the incidence of diarrhea and flatulence was low and there were no adverse event reports of constipation. The control group showed fewer formed and more loose stools as compared to the test group. This can be explained by the previously reported greater stool softening effect of 0.8 g/100 mL vs. 0.4 g/100 mL scGOS/lcFOS. Moro et al. [29] compared a formula without scGOS/lcFOS to a formula containing 0.4 g/100 mL scGOS/lcFOS (i.e., the level in the test product) and a formula containing 0.8 g/100 mL scGOS/lcFOS (i.e., the level in the control product) and concluded that scGOS/lcFOS resulted in softer stools in a dose-dependent manner, with a dosage of 0.8 g/100 mL resulting in softer stools compared to a dosage of 0.4 g/100 mL [29]. The median daily stool frequency observed in the study population was comparable to the stool frequencies reported in studies in healthy infants fed with formulae containing prebiotics and/or postbiotics [30,31,32].

The average baseline IGSQ sum score of the study population was almost 32 points. Based on the literature, this score can be considered to reflect a relevant GI symptom burden [20]. Regurgitation is often associated with other GI symptoms, such as infant colic, gas/bloating, and constipation [4,5,6]. Throughout the intervention, IGSQ sum scores decreased to scores of <25, which is in line with scores observed in healthy infants without specific GI issues [33,34,35,36,37]. The decrease in the IGSQ sum scores observed in the current trial can therefore be considered a meaningful improvement of GI burden. The improved IGSQ sum score in the control group was not unexpected as scGOS/lcFOS and postbiotics in infant formulae are reported to have beneficial effects on gastrointestinal outcomes such as stool consistency [30,38,39]. In addition, reassurance of by the parents and the natural evolution of regurgitation might have contributed to the improved scores throughout the study [10].

International recommendations refer to reassurance by the parents and dietary advice, including the use of thickened AR formulae in children who are not fully breastfed [9,10]. The current study showed that infants receiving the AR formula with LBG combined with the prebiotics, scGOS/lcFOS (9:1), and postbiotics derived from the Lactofidus^TM^ process resulted in a significantly larger reduction of regurgitation frequency compared to the control formula without LBG. Moreover, the parent-reported outcomes indicated that the formula helped to reduce regurgitation and positively affected the well-being of their baby.

The current study was designed as an RCT to provide solid evidence on the gastrointestinal tolerance and safety of locust bean gum as a thickening agent in infant formulae. The study responds to a specific requirement from the EFSA and stool consistency was considered a relevant and reliable indicator for gastrointestinal tolerance [20]. The study complements the results of earlier investigations in real-world settings and of a recent double-blind RCT with the AR formula (containing LBG and pre- and/or postbiotics) in infants with regurgitation [14,17,18].

One of the study limitations relates to the baseline regurgitation frequency. In the current study it was somewhat lower (median of 3.5 episodes/day in the test group; 3.0 episodes/day in the control group) than the frequencies reported in several other studies in infants with regurgitation (e.g., 3.7 episodes/day [40]), and as recently reviewed [2]. However, it aligns with the Rome IV criteria for functional regurgitation and with three previous reports showing that even a frequency of regurgitating of twice a day may cause parental concern with 20–40% of the mothers asking for medical advice for this symptom [41,42,43]. Since parental concern increases with the frequency and volume of regurgitation, particularly when more than three episodes occur per day [41,42], we performed a sub-analysis of these infants for a possible more relevant clinical significance. The subgroup of infants presenting frequent regurgitation per day and who received the test product showed a larger reduction of regurgitation episodes compared to the control group. In addition, the current study was primarily developed as a tolerance and safety study and powered to study non-inferiority of stool consistency. A secondary study aim was to investigate the efficacy of the AR formula by studying the effect on regurgitation. Infants were selected based on the adapted Rome IV criteria for the diagnosis of regurgitation, meaning the presence of ≥two episodes of regurgitation per day for ≥1 week prior to screening instead of at least 3 weeks for diagnosing regurgitation [21]. This was carried out for feasibility reasons, as infants with a longer history or regurgitation were expected to have already been using (other) AR formula or other reflux treatments, which were not allowed in this study. The selection of infants based on the adapted Rome IV criteria might have contributed to the relatively low baseline regurgitation frequency. Nevertheless, the average duration of regurgitation prior to inclusion in the study was 25.6 days and regurgitation frequency decreased to a similar extent in the test group as compared to the effects observed in earlier AR efficacy studies [2].

Another limitation of this RCT is that it could not be labelled as double-blind because of the small chance that the nature of the study product, due to difference between the test and control product in viscosity, would be noticed by the parents. However, all measures were taken to maintain the blinding and all procedures were followed as for a double-blind RCT. In addition, although subjects did drop out from the study, the number was not significantly different between the two groups and was low considering that part of the study was performed during the COVID-19 pandemic. Finally, blood samples for nutritional and mineral assessments were only available for four infants. However, it would be unethical and not in line with the Rome IV criteria and the guidelines on gastro-esophageal reflux to consider blood collection mandatory for infants with functional regurgitation.

## 5. Conclusions

This study among infants with functional regurgitation showed that the test AR formula containing LBG combined with the prebiotic mixture scGOS/lcFOS (9:1) and postbiotics derived from the Lactofidus^TM^ process did not lead to more loose or watery stools than the control formula with scGOS/lcFOS (9:1) and postbiotics, but without LBG. Both products were well-tolerated and safe. The study provides further confirmation on the significant beneficial effect of the infant formula containing LBG combined with pre- and postbiotics on the dietary management of regurgitation in infants who are not exclusively breastfed, and the reassurance it can bring to parents.

## Data Availability

The data presented in this study are available on request. Data requests can be directed to datasharing.clinicalresearchnutricia@danone.com. Decisions regarding the availability of the data will be made on a case-by-case basis, considering the purpose, context of use, and compliance with relevant privacy and ethical regulations.

## Supplementary Materials

The following supporting information can be downloaded at: https://www.mdpi.com/article/10.3390/nu16060899/s1, Table S1: Main nutritional components of study products; Table S2: Demographic and baseline characteristics of infants per study group; Table S3: Infant Gastrointestinal Symptom Questionnaire (IGSQ) item scores.
