# Supplementary material for: Tolerance and Safety of an Anti-Regurgitation Formula Containing Locust Bean Gum, Pre-, and Postbiotics: A Multi-Country Multi-Center Prospective Randomized Controlled Study in Infants with Regurgitation
Source: Nutrients. 2024 Mar 20;16(6):899. doi: 10.3390/nu16060899 (PMC10974053; doi:10.3390/nu16060899)
Supplement: Supplementary file 1 [file nutrients-16-00899-s001.zip › nutrients-2865811-supplementary.pdf]

nutrients

MDPI

Supplementary Materials

Table S1: Main nutritional components of study products

	Test formula[1]	Control formula[2]
**Parameter**	**Prepared per 100 mL**	**Prepared per 100 mL**
**Energy (kcal)**	66	66
**Protein (g)**	1.3	1.3
**Carbohydrates (g)**	7.3	7.4
**Fats (g),** of which:	3.4	3.4
saturated fatty acids	1.5	1.5
**Non-digestible carbohydrates / fiber (g)**		
scGOS [#]	0.36 (fiber: 0.24)	0.72 (fiber: 0.48)
lcFOS	0.04	0.08
LBG	0.4	-
**Partly fermented formula with postbiotics** derived from Lactofidus™ fermentation process	26%	26%

[#] 67% of scGOS in EC declaration can be considered fiber

[1]Contents of minerals and vitamins are based on EU regulation for Food for Special Medical Purposes (2016/128/EC)

[2]Contents of minerals and vitamins are based on EU regulation 2016/127/EC.

Table S2: Demographic and baseline characteristics of infants per study group in the All-Subjects-Randomised population

	**Test group**<br>n=51	**Control group**<br>n=52
Infant sex, % male/female	37.3/62.7	50.0/50.0
Race, n (% of subjects)		
White	50 (100.0%)	49 (98.0%)
Other	0 (0.0%)	1 (2.0%)
Missing	1	2
Gestational age (days), median (Q1-Q3)	277 (272; 280)	273 (271; 280)
Age at inclusion (days), mean (SD)	37.3 (13.0)	36.4 (12.8)
Country (% Poland/Ukraine/Italy)	58.8/31.4/9.8	63.5/26.9/9.6
Birth weight (g), median (Q1-Q3)	3480 (3100; 3750)	3300 (3115; 3645)
Birth length (cm), median (Q1-Q3)	54 (51; 56)	53 (51; 56)
Maternal age (years; mean ± SD)	29.7 (6.3)	28.5 (5.0)
Mode of delivery, % vaginal/c-section	51.0/49.0	50.0/50.0
Duration of regurgitation prior to inclusion (days), mean (SD)	26.0 (12.0)	25.2 (10.7)
Duration of exclusive formula feeding prior to inclusion (days), mean (SD)	29.1 (15.0)	26.1 (14.2)
Type of formula used prior to inclusion, n (% of subjects)		
Formula containing <0.5 g/100 ml prebiotics	33 (64.7%)	20 (38.5%)
Formula containing 0.5-0.8 g/100 ml prebiotics	27 (52.9%)	37 (71.2%)

Table S3 : Infant Gastrointestinal Symptom Questionnaire (IGSQ) item scores in the Per-Protocol population

**Parameter**	**Test group** n=48			**Control group** n=52			**p-value**[1]
	n	Mean ± SD	Median (Q1; Q3)	n	Mean ± SD	Median (Q1; Q3)	
**IGSQ item 1:** Thinking about the past week, how many times did your baby pass a hard stool?							
Baseline (Visit 1)	48	2.0 (1.2)	1 (1; 3)	52	1.8 (1.2)	1 (1; 2)	0.312
Week 2 (Visit 2)	44	2.0 (1.1)	1 (1; 3)	51	1.4 (0.9)	1 (1; 1)	0.008
Week 4 (Visit 3)	44	1.7 (1.0)	1 (1; 2)	51	1.4 (0.7)	1 (1; 1)	0.072
Week 8 (Visit 4)	43	1.4 (0.7)	1 (1; 2)	50	1.2 (0.5)	1 (1; 1)	0.237
Age of 17 weeks (Visit 5)	37	1.2 (0.5)	1 (1; 1)	42	1.2 (0.4)	1 (1; 1)	0.741
**IGSQ item 2:** Thinking about the past week, how many times did your baby have difficulty when passing a bowel movement?							
Baseline (Visit 1)	48	2.4 (1.3)	3 (1; 3)	52	2.6 (1.3)	3 (1; 4)	0.275
Week 2 (Visit 2)	44	2.2 (1.1)	2 (1; 3)	51	1.8 (0.9)	2 (1; 3)	0.161
Week 4 (Visit 3)	44	1.9 (1.1)	2 (1; 3)	51	1.6 (1.0)	1 (1; 2)	0.167
Week 8 (Visit 4)	43	1.6 (0.9)	1 (1; 2)	50	1.3 (0.6)	1 (1; 1)	0.121
Age of 17 weeks (Visit 5)	37	1.3 (0.5)	1 (1; 2)	42	1.2 (0.4)	1 (1; 1)	0.252
**IGSQ item 3:** Thinking about a usual day in the past week, how many times did milk come out of your baby's mouth?							
Baseline (Visit 1)	48	3.9 (0.7)	4 (3; 4)	52	3.6 (0.8)	4 (3; 4)	0.071
Week 2 (Visit 2)	44	2.3 (0.8)	2 (2; 3)	51	2.7 (0.8)	3 (2; 3)	0.055
Week 4 (Visit 3)	44	1.9 (0.8)	2 (1; 3)	51	2.5 (0.9)	3 (2; 3)	0.001
Week 8 (Visit 4)	43	1.5 (0.6)	1 (1; 2)	50	2.2 (1.0)	2 (1; 3)	<.001
Age of 17 weeks (Visit 5)	37	1.6 (0.9)	1 (1; 2)	42	2.1 (0.8)	2 (2; 3)	0.004

**IGSQ item 4:** Thinking about the past week, how much milk usually came out each time?							
Baseline (Visit 1)	48	1.7 (0.7)	2 (1; 2)	52	1.8 (0.8)	2 (1; 2)	0.997
Week 2 (Visit 2)	44	1.3 (0.6)	1 (1; 2)	51	1.3 (0.6)	1 (1; 2)	0.774
Week 4 (Visit 3)	44	1.1 (0.3)	1 (1; 1)	51	1.2 (0.5)	1 (1; 1)	0.180
Week 8 (Visit 4)	42	1.0 (0.2)	1 (1; 1)	50	1.2 (0.4)	1 (1; 1)	0.088
Age of 17 weeks (Visit 5)	37	1.0 (0.2)	1 (1; 1)	42	1.1 (0.3)	1 (1; 1)	0.128
**IGSQ item 5:** Thinking about the past week, how often did your baby seem uncomfortable or fussy when milk came out of his or her mouth?							
Baseline (Visit 1)	48	2.5 (1.1)	3 (1; 3)	52	2.3 (1.2)	2 (1; 3)	0.449
Week 2 (Visit 2)	44	1.8 (0.9)	2 (1; 3)	51	1.8 (1.0)	1 (1; 3)	0.901
Week 4 (Visit 3)	44	1.7 (0.9)	1 (1; 3)	51	1.5 (0.8)	1 (1; 2)	0.623
Week 8 (Visit 4)	43	1.3 (0.7)	1 (1; 1)	50	1.4 (0.8)	1 (1; 1)	0.681
Age of 17 weeks (Visit 5)	37	1.1 (0.4)	1 (1; 1)	42	1.2 (0.4)	1 (1; 1)	0.497
**IGSQ item 6:** Thinking about the past week, how many times did your baby arch his or her back as if in pain when milk came out of his or her mouth?							
Baseline (Visit 1)	48	2.2 (1.4)	2 (1; 3)	51	2.2 (1.3)	2 (1; 3)	0.988
Week 2 (Visit 2)	44	1.5 (0.8)	1 (1; 2)	51	1.5 (0.9)	1 (1; 2)	0.861
Week 4 (Visit 3)	44	1.3 (0.6)	1 (1; 1)	51	1.4 (0.7)	1 (1; 2)	0.148
Week 8 (Visit 4)	43	1.2 (0.5)	1 (1; 1)	50	1.2 (0.4)	1 (1; 1)	0.786
Age of 17 weeks (Visit 5)	37	1.0 (0.2)	1 (1; 1)	42	1.1 (0.3)	1 (1; 1)	
**IGSQ item 7:** Thinking about the past week, how much total time did your baby usually cry in a day?							
Baseline (Visit 1)	48	2.3 (1.1)	2 (2; 3)	52	2.3 (1.2)	2 (2; 3)	0.726
Week 2 (Visit 2)	44	1.7 (0.8)	2 (1; 2)	51	1.7 (0.9)	2 (1; 2)	0.890
Week 4 (Visit 3)	44	1.5 (0.6)	1 (1; 2)	50	1.7 (0.8)	2 (1; 2)	0.105
Week 8 (Visit 4)	43	1.3 (0.6)	1 (1; 1)	50	1.5 (0.7)	1 (1; 2)	0.154
Age of 17 weeks (Visit 5)	37	1.4 (0.7)	1 (1; 2)	42	1.3 (0.8)	1 (1; 1)	0.364

**IGSQ item 8:** Thinking about the past week, how many times were you unable to soothe your baby to stop his or her crying?							
Baseline (Visit 1)	48	2.0 (1.3)	1 (1; 3)	52	1.8 (1.2)	1 (1; 2)	0.451
Week 2 (Visit 2)	44	1.8 (1.0)	1 (1; 2)	51	1.6 (1.0)	1 (1; 2)	0.483
Week 4 (Visit 3)	44	1.4 (0.7)	1 (1; 2)	51	1.5 (1.0)	1 (1; 2)	0.988
Week 8 (Visit 4)	43	1.4 (1.0)	1 (1; 1)	50	1.4 (0.9)	1 (1; 1)	0.996
Age of 17 weeks (Visit 5)	37	1.2 (0.8)	1 (1; 1)	42	1.3 (0.8)	1 (1; 1)	0.357
**IGSQ item 9:** Thinking about the past week, how many times did your baby cry during or right after a feeding because the milk bothered your baby?							
Baseline (Visit 1)	48	2.3 (1.5)	2 (1; 4)	52	2.1 (1.4)	1 (1; 3)	0.674
Week 2 (Visit 2)	44	1.7 (1.1)	1 (1; 2)	51	1.8 (1.2)	1 (1; 3)	0.782
Week 4 (Visit 3)	44	1.6 (0.9)	1 (1; 2)	51	1.8 (1.1)	1 (1; 2)	0.524
Week 8 (Visit 4)	43	1.5 (1.1)	1 (1; 1)	50	1.2 (0.4)	1 (1; 1)	0.254
Age of 17 weeks (Visit 5)	37	1.4 (0.9)	1 (1; 1)	42	1.3 (0.6)	1 (1; 1)	0.937
**IGSQ item 10:** Thinking about the past week, on how many days was your baby fussy?							
Baseline (Visit 1)	48	2.6 (1.0)	3 (2; 3)	52	2.8 (1.3)	3 (1; 4)	0.525
Week 2 (Visit 2)	44	2.3 (0.9)	2 (2; 3)	51	2.3 (1.0)	2 (1; 3)	1.000
Week 4 (Visit 3)	44	2.0 (1.0)	2 (1; 3)	51	2.1 (0.9)	2 (1; 3)	0.661
Week 8 (Visit 4)	43	1.9 (1.0)	2 (1; 2)	50	1.7 (1.1)	1 (1; 2)	0.283
Age of 17 weeks (Visit 5)	37	1.7 (1.0)	1 (1; 2)	42	1.8 (1.1)	1 (1; 2)	0.987
**IGSQ item 11:** Thinking about the past week, how many times were you unable to soothe your baby when he or she was fussy?							
Baseline (Visit 1)	48	1.9 (1.3)	1 (1; 2)	52	1.9 (1.4)	1 (1; 3)	0.804
Week 2 (Visit 2)	44	1.5 (0.8)	1 (1; 2)	51	1.6 (0.9)	1 (1; 2)	0.695
Week 4 (Visit 3)	44	1.4 (0.7)	1 (1; 2)	51	1.5 (0.9)	1 (1; 2)	0.689
Week 8 (Visit 4)	43	1.4 (0.9)	1 (1; 1)	50	1.4 (0.9)	1 (1; 1)	0.719
Age of 17 weeks (Visit 5)	37	1.2 (0.7)	1 (1; 1)	42	1.4 (0.8)	1 (1; 1)	0.291

**IGSQ item 12:** Thinking about the past week, how many times in a usual day was your baby gassy?							
Baseline (Visit 1)	48	4.0 (0.9)	4 (3; 5)	52	4.1 (0.9)	4 (3; 5)	0.459
Week 2 (Visit 2)	44	3.7 (1.1)	4 (3; 5)	51	3.7 (1.3)	4 (3; 5)	0.966
Week 4 (Visit 3)	44	3.5 (1.2)	3 (3; 5)	51	3.7 (1.4)	4 (3; 5)	0.287
Week 8 (Visit 4)	43	3.3 (1.3)	3 (2; 5)	50	3.6 (1.4)	4 (2; 5)	0.473
Age of 17 weeks (Visit 5)	37	3.1 (1.4)	3 (2; 4)	42	3.2 (1.5)	4 (2; 5)	0.673
**IGSQ item 13:** Thinking about the past week, how often did gas seem to make your baby uncomfortable or fussy?							
Baseline (Visit 1)	48	2.4 (1.3)	3 (1; 4)	52	2.6 (1.2)	3 (1; 3)	0.484
Week 2 (Visit 2)	44	1.9 (1.0)	2 (1; 3)	51	2.1 (1.1)	2 (1; 3)	0.554
Week 4 (Visit 3)	44	2.0 (1.0)	2 (1; 3)	51	1.8 (1.0)	1 (1; 3)	0.451
Week 8 (Visit 4)	43	1.7 (0.8)	1 (1; 2)	50	1.6 (0.8)	1 (1; 2)	0.698
Age of 17 weeks (Visit 5)	37	1.3 (0.6)	1 (1; 1)	42	1.3 (0.6)	1 (1; 1)	0.814

[1] p-value based on Mann-Whitney U test between test and control group.
